# Left Atrial Myxoma Presenting With Non-ST Segment Elevation Myocardial Infarction

**DOI:** 10.7759/cureus.78178

**Published:** 2025-01-29

**Authors:** Priyadarshini Dixit, Shivani Mehta, Sharvil Patel, Vikas Kilaru, Alex M Adams

**Affiliations:** 1 Graduate Medical Education, Northeast Georgia Medical Center Gainesville, Gainesville, USA; 2 Cardiovascular Disease, Northeast Georgia Medical Center Gainesville, Gainesville, USA; 3 Internal Medicine, Northeast Georgia Medical Center Gainesville, Gainesville, USA

**Keywords:** left atrial myxoma, myxomatous embolization, new-onset cardiomyopathy, non-obstructive coronary artery disease, non-st segment elevation myocardial infarction (nstemi)

## Abstract

Cardiac myxoma is a benign cardiac tumor. A rare phenomenon associated with cardiac tumors is the embolization of the myxomatous material into the coronary arteries, leading to myocardial infarction. This typically requires surgical excision of the tumor to prevent recurrence. Early diagnosis with the help of both transthoracic and transesophageal echocardiography is vital in managing these patients. Here, we present a case of a 49-year-old male patient who initially presented with chest pain and non-ST segment elevation myocardial infarction (NSTEMI) and subsequently newly diagnosed cardiomyopathy. Upon further cardiac evaluation, he was found to have a large left atrial myxoma with non-obstructive coronary artery disease. It was suspected that the etiology for NSTEMI and subsequent newly diagnosed cardiomyopathy was due to myxomatous embolization into the coronary arteries; ischemic cardiomyopathy was excluded by definition. The patient subsequently underwent successful surgical excision of the left atrial myxoma.

## Introduction

Myxomas represent the predominant type of cardiac tumor, accounting for 50% of all primary cardiac tumors [1]. They predominantly arise from the endocardial tissue of the atria, particularly the left atrium. Cardiac myxomas are most commonly diagnosed between the fourth and sixth decades of life and are more frequently observed in women [2]. Although considered benign, they can manifest with severe cardiovascular complications due to their strategic location and size. Symptoms vary and depend on the size and location. Complications include obstruction leading to heart failure and systemic embolization to distal tissue [3]. Constitutional symptoms include fever, malaise, and weight loss. We present to you a case of a patient with myxomatous embolization, which is a rare phenomenon complicated by non-ST segment elevation myocardial infarction (NSTEMI) and was treated successfully with surgical excision.

## Case presentation

**Figure 1:**
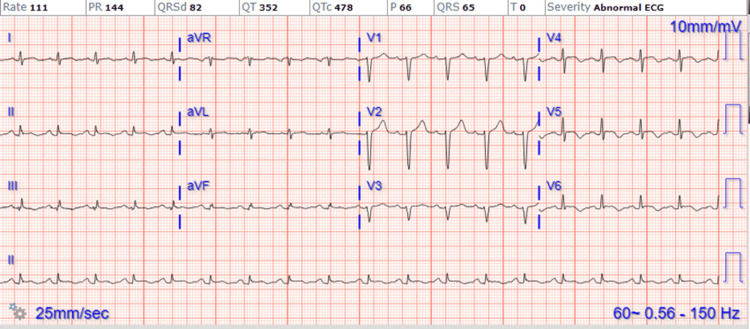
EKG demonstrating sinus tachycardia with T-wave inversions in the lateral leads

A 49-year-old male patient with a past medical history of diabetes mellitus, hypertension, hyperlipidemia, and former tobacco abuse presented to our facility with complaints of chest pain lasting one day. The chest pain was intermittent, mid-sternal in nature, radiated to his back, and was associated with nausea and vomiting. The patient denied any history of aggravating or relieving factors and reported no similar symptoms in the past. The physical examination revealed normal heart sounds, no murmurs, and no pedal edema. An electrocardiogram (EKG) revealed sinus tachycardia with T-wave inversion in the lateral leads (Figure 1). The initial significant laboratory investigations are provided in Table 1.

**Table 1:** Significant laboratory investigations

Parameters	Value	Reference Range
Hemoglobin	16.6 g/dL	13-18 g/dL
Platelet count	347 x 10^3^/mL	150-400 x 10^3^/mL
Sodium	137 mmol/L	136-145 mmol/L
Potassium	4.0 mmol/L	3.6-5.2 mmol/L
Creatinine	0.97 mg/dL	0.6-1.2 mg/dL
High-sensitivity troponin	17,000 ng/L	<14 ng/L

CT angiography ruled out aortic dissection and the presence of an aneurysm. Given the clinical presentation was concerning for NSTEMI type I, the patient was started on IV heparin infusion per acute coronary syndrome (ACS) protocol. He was also started on aspirin, a high-intensity statin, beta-blockers, and nitroglycerin as needed.

Transthoracic echocardiography (TTE) revealed a left ventricular ejection fraction of 30-35% with severe global hypokinesis. Trace mitral regurgitation and a large echo density in the left atrium attached to the interatrial septum were also noted. Transesophageal echocardiography (TEE) was performed to investigate and delineate the TTE findings further. TEE revealed a 4 x 2.8 cm large, mobile, and heterogeneous irregular mass present in the interatrial septum consistent with the tumor and no associated significant valvular pathology (Videos 1, 2).

**Video 1:** Four-chamber view of TEE demonstrating left atrial mass attached to the interatrial septum

**Video 2:** 3D TEE demonstrating left atrial mass

In the presence of NSTEMI and newly reduced ejection fraction, left heart catheterization with coronary angiography was performed to exclude obstructive coronary artery disease. Cardiac catheterization demonstrated normal epicardial coronary arteries with no evidence of obstructive disease. The etiology for cardiac myocardial injury and tissue damage leading to troponin elevation was suspected to be due to myxomatous embolization in the setting of no acute ECG changes and normal epicardial coronary arteries. A multidisciplinary team approach was taken, which involved CT surgery, anesthesia, and cardiology teams. The patient had a low surgical risk for cardiac surgery and surgical excision.

Informed consent was obtained, and the patient underwent successful robotic-assisted left atrial mass resection. The patient tolerated the procedure well and was monitored overnight in the cardiac critical care unit. An excised tissue sample was sent to the pathology laboratory for tissue examination, which confirmed the atrial mass as a benign myxoma.

## Discussion

The etiology of atrial myxomas is still under investigation; however, immunohistochemical studies suggest that most myxoma cells originate from multipotent mesenchymal cells capable of both neural and endothelial dysfunction [4]. Myxomas are often characterized as pedunculated and gelatinous with a smooth or friable surface, and their diameter can range from as small as 1 cm up to 15 cm [5,6]. Although considered benign, they can manifest with severe cardiovascular complications due to their strategic location.

Individuals with cardiac myxomas can present with a wide variety of clinical manifestations, ranging from constitutional symptoms of fever, malaise, and weight loss to sudden cardiac death. Constitutional symptoms are related to the overproduction of interleukin 6, which plays a major role in the proliferation of myxoma cells [5,6]. Left atrial myxomas can mimic signs and symptoms of valvular heart disease or heart failure, such as dyspnea with exertion, orthopnea, paroxysmal nocturnal dyspnea, and pulmonary edema [7]. Symptom manifestations depend on the size, mobility, and location of the tumor. The most severe complication of myxomas is embolization, occurring in approximately 30-40% of patients [7]. Left-sided atrial myxomas commonly embolize to cerebral and retinal arteries, causing ischemic stroke and visual loss [8]. Embolization can also occur in the coronary, renal, visceral, and abdominal arteries [9]. Studies have linked higher platelet counts and irregular surface characteristics to an increased risk of embolization [9]. Due to the high risk of embolism, prompt surgical resection is often indicated after diagnosis.

Special considerations pre- and post-surgically should be made for atrial myxomas of substantial size. They typically range in size from 0.4 to 6.5 cm [10]. Preoperative planning should include considerations of complete surgical excision and care not to damage proximal cardiac structures, for which cardiac MRI or cardiac CT can be utilized for enhanced visualization and characterization. Friable appearing tissues may be an indicator of concomitant embolic events or varying histopathological profiles, with special consideration for vascularity, heterogeneity in tissue makeup, and degree of necrosis and degeneration [11]. Such deliberations could provide insight into surgical techniques, prognosis, and long-term follow-up planning. Although metastasis in primary cardiac tumors is rare, increased vigilance is necessary for the potential of retained tissue, satellite lesions, and incomplete resection in exceptionally sizable masses [12].

## Conclusions

Although myxoma is one of the most common benign tumors in the heart, its clinical presentation can be in various forms, ranging from constitutional symptoms to even cardiac death. One of the rare phenomena caused by a large myxoma is myxomatous embolization, which can result in stroke, sudden cardiac death, and coronary artery embolization, presenting as acute myocardial infarction. Early diagnosis with the help of imaging modalities such as TTE and TEE before coronary angiography is of utmost importance to rule out other etiologies of presenting clinical symptoms. Our patient presented with NSTEMI due to myxomatous embolization of the myxoma, which was later successfully excised due to early recognition and diagnosis.
